# The association between individual radiographic findings and improvement after chiropractic spinal manipulation and home exercise among older adults with back-related disability: a secondary analysis

**DOI:** 10.1186/s12998-024-00566-9

**Published:** 2025-01-07

**Authors:** Michele J. Maiers, Andrea K. Albertson, Christopher Major, Heidi Mendenhall, Christopher P. Petrie

**Affiliations:** 1 Northwestern Health Sciences University, 2501 W 84th Street, Bloomington, MN 55431 USA; 2 RAND Research Across Complementary and Integrative Health Institutions (REACH) Center, 1776 Main Street, Santa Monica, CA 90401-3208 USA

**Keywords:** Spinal manipulation, Home exercise, Radiography, Back pain, Older adults, Disability, Degeneration, Alignment, Responder, Chiropractic

## Abstract

**Background:**

Some chiropractors use spinal x-rays to inform care, but the relationship between radiographic findings and outcomes is unclear. This study examined the association between radiographic findings and 30% improvement in back-related disability in older adults after receiving 12 weeks of chiropractic spinal manipulation and home exercise instruction.

**Methods:**

This IRB-approved secondary analysis used randomized trial data of community-dwelling adults age *≥* 65 with chronic spinal pain and disability. Data were collected during the parent trial between January 2010-December 2014. The primary outcome of the parent study was ≥ 30% improvement in Oswestry Disability Index (ODI) at 12 weeks, a clinically important response to care. In this secondary analysis, two chiropractic radiologists independently assessed digital lumbar radiographs for pre-specified anatomic, degenerative, and alignment factors; differences were adjudicated. The unadjusted association between baseline radiographic factors and 30% ODI improvement was determined using chi-square tests.

**Results:**

From the parent trial, 120 adults with baseline lumbar radiographs were included in this study. Mean age was 70.4 years (range 65–81); 59.2% were female. Mean baseline disability (ODI = 25.6) and back pain (5.2, 0–10 scale) were moderate. Disc degeneration (53.3% moderate, 13.3% severe), anterolisthesis (53.3%), retrolisthesis (36.6%) and scoliosis (35.0%) were common among the participant sample. After 12-weeks of treatment, 51 (42.5%) participants achieved 30% improvement in back disability. No alignment, degenerative, or anatomic factors were associated with ODI improvement at 12 weeks (all *p* > 0.05), regardless of severity of radiographic findings.

**Conclusion:**

We found no association between a predetermined subset of radiographic findings and improvement in back-related disability among this sample of older adults. As such, this study provides preliminary data suggesting that imaging may be unhelpful for predicting response to chiropractic spinal manipulation and home exercise.

**Supplementary Information:**

The online version contains supplementary material available at 10.1186/s12998-024-00566-9.

## Introduction

Individuals age 65 and older comprise an increasingly large proportion of the population among high-income nations [1]. Low back pain is highly prevalent among older adults, affecting 21–75% annually [2]. Nonsurgical, nonpharmacologic approaches to care are of particular interest for older adults with spine-related pain and disability, given their increased risk of complications from pharmacologic interventions and spine surgery [3–6].

Most spine-related complaints in seniors have no definite pathology, making them potentially amenable to chiropractic treatment and other nonoperative approaches [7, 8]. There is growing evidence that spinal manipulation and exercise, commonly prescribed by Doctors of Chiropractic, reduces pain and pain-related disability in older adults [9–13]. Recent studies estimate that adults ages 65 or older account for 14–16% of adult chiropractic users, and this proportion is increasing over time [14–18]. 

Spinal radiographs may aid the clinical examination and diagnosis of spine conditions. For providers who perform spinal manipulation, radiographs also identify pathologic conditions that are known or thought to be contraindications to manipulation, such as fractures, malignancies, severe osteoporosis, or abdominal aortic aneurysm [19]. Historically and anecdotally, chiropractors have additionally used spinal radiographs to inform segmental spinal manipulation decisions, care management plans, or to determine who may respond to spinal manipulation [20, 21]. The extent to which this is currently practiced is not well described in the literature [22]. Moreover, there is a paucity of research supporting the use of imaging for these purposes. One observational study of over 2,000 adult chiropractic patients with low back pain found that diagnostic imaging did not result in better clinical outcomes [23]. Given potential risks associated with unnecessary imaging, including radiation, cost, and unnecessary pursuit of incidental findings, it is important to more thoroughly explore imaging practices for older adult patients [24]. The association between baseline radiographic findings and clinical outcomes among older adult chiropractic users is unknown. Specifically, it has not been determined whether baseline radiographic findings help predict early pain and functional response to chiropractic spinal manipulation and home exercise in older adults with back and neck pain.

The goal of this study was to examine the association between anatomic, degenerative, and anatomical radiographic findings commonly noted in chiropractic practice, and 30% improvement in back-related disability in older adults after 12 weeks of chiropractic spinal manipulation and home exercise instruction.

## Methods

This retrospective cohort study is a secondary analysis of randomized clinical trial (RCT) data collected on community-dwelling adults ages 65 years or older with chronic spinal pain and disability, and was approved by the Institutional Review Board at Northwestern Health Sciences University [25–27]. Recruitment and data collection for the parent RCT was conducted from January 2010 through December 2014. All participants enrolled in the parent RCT received the same study intervention, consisting of primarily high-velocity, low-amplitude chiropractic spinal manipulation plus home exercise instruction for 12 weeks [26]. In this secondary study, we included adults who had baseline radiographs taken of the lumbar spine to determine eligibility for the parent trial, which excluded individuals with advanced spinal stenosis, fracture, or severe osteoporosis. Routine imaging for spinal pain in older adults was considered a best practice the time of the study. Participants who did not have radiographs taken at baseline had advanced imaging within one year prior to trial enrollment, and were therefore not required to have lumbar radiographs in the absence of new clinical symptoms [25]. Those participants were excluded from this study.

Participant demographic information, as well as pain and functional measures collected in the parent RCT, were also included in this analysis. The primary outcome of the parent study was a clinically significant change in disability, considered to be *≥* 30% improvement in Oswestry Disability Index (ODI) after 12 weeks of study intervention [28]. These results are reported elsewhere [27]. 

In this study, a team of three chiropractic radiologists and two researchers (also chiropractors with clinical experience) agreed on radiographic findings of the lumbar spine conventionally considered in the chiropractic profession to be of possible clinical importance, including anatomic, degenerative, and alignment factors [29, 30]. Methods for assessing and scoring them were identified based on the literature and common practice among chiropractic radiologists (Appendix 1) [31–42]. These methods were further pilot tested by two chiropractic radiologists, who independently assessed a subsample of digital lumbar radiographs. Their experience was used to refine the methods of standardization used by the radiologists in this study.

Once the methodology to identify radiographic factors was finalized, two rounds of radiographic readings were undertaken by study radiologists (HM, CM) on the images of 10 study participants each. These were independently evaluated, with the radiologists convening after each round to consult with one another to identify agreement, discuss differences, and reach consensus on findings as needed. This process helped ensure assessment protocols were suitable for the remainder of the analysis, which followed. Findings were independently entered into Excel spreadsheets. Once completed, the study coordinator (AA) compared radiologists’ findings and checked scoring for consistency. Discrepancies were identified and presented to the radiologists for discussion and consensus. A third radiologist (CP) was available to adjudicate disagreements.

Descriptive baseline statistics are reported for this subsample of the parent RCT. Analysis for this study includes assessing the unadjusted association between individual baseline radiographic factors and 30% ODI improvement with unadjusted logistic regression. Analyses were conducted using SAS 9.4^®^ software.

## Results

Of 182 participants in the parent trial, 120 (66%) had baseline lumbar radiographs with complete baseline and 12-week data and were therefore included in this study. Mean participant age was 70.4 years (range 65–81) and 59.2% were female (Table 1). Mean baseline back-related disability (ODI = 25.6) and back pain (5.2, 0–10 scale) were moderate, and 40% of adults reported some leg pain at baseline. The most common radiographic findings are reported in Table 2, and included disc degeneration (53.3% moderate, 13.3% severe), anterolisthesis (53.3%), retrolisthesis (36.6%) and scoliosis (35.0%).

**Table 1:** Baseline characteristics of participants

Baseline characteristics	Overall N = 120 n (%)
Demographic	
Mean age (sd)	70.4 (4.7)
Age 70 or older	55 (45.8)
Female	71 (59.2)
White race	114 (95.0)
Lifestyle choices	
BMI, mean (sd)	28.6 (5.9)
Tobacco use (any)	10 (8.3)
Average weekly exercise: *2–3 times/week or more*	73 (60.8)
Amount of physical activity in daily routine: ≥ *moderate*	65 (54.2)
Low back status	
Low back pain duration, years (median, IQR)	15.0 (5–30)
Low back pain severity: mean, past week (0≥10)	5.2 (2.2)
Low back pain severity ≥ 5.0	54 (45.0)
Low back pain + any leg pain (QTF ≥ 2, range 2–4)	48 (40.0)
Leg pain severity past week (0–10) mean (sd)	3.1 (2.2)
Back-related disability (Oswestry Disability Index) mean (sd) (0-100)	25.6 (9.6)
Function	
Short Performance Physical Battery (SPPB), mean (sd)	8.6 (1.8)
SPPB < 10 (at least 1 mobility limitation)	76 (63.3)
Psychosocial	
Geriatric Depression Scale (GDS), mean, (median)	2.0 (2.2)

sd = standard deviation; BMI = Body Mass Index; IQR = interquartile range; QTF = Quebec Task Force

**Table 2:** Radiographic findings in RCT participants with lumbar spine x-rays

Variable	*N* = 120 *n* (%)
*Coronal measures*	
Scoliosis (> 10 degrees)	42 (35.0)
Mean Cobb angle (sd)	17.3 (6.5)
Scoliosis levels	
L1-L5	11/42 (24.4)
L2-L5	11/42 (24.4)
L1-L4	8/42 (17.8)
T12-L5	3/42 (6.7)
Other	9/42 (21.4)
Trunk shift (≥ 2 cm)	4 (3.3)
*Sagittal measures*	
Lumbar lordosis (mean, sd)	52.0 (12.1)
Sacral base angle (mean, sd)	35.2 (8.6)
Ferguson’s weight bearing line*	
Normal	44 (36.7)
Anterior	56 (46.7)
Posterior	20 (16.7)
A or P weightbearing alteration	76 (63.3)
Anterolisthesis (any)	64 (53.3)
One level	53/64 (82.8)
Two levels	11/64 (17.2)
Maximal anterior translation level:	
L4 on L5	40/64 (62.5)
L5 on S1	14/64 (21.9)
Other	10/64 (15.6)
Millimeters of slip** (mean, sd)	3.3 (0.8)
Meyerding classification*	
Grade I	63/64 (98.4)
Grade II	1/64 (1.6%)
Wiltse-Newman type	
Type 3	55/64 (85.9)
Type 2	9/64 (14.1)
Retrolisthesis (any)	44 (36.6)
Vertebral wedging	17 (14.2)
None	103 (85.8)
1 level	15 (12.5)
2 levels	2 (1.7)
Disc degeneration	
Any level(s), severe	16 (13.3)
Any level(s), moderate	64 (53.3)
Any level(s), mild	107 (89.2)
Any disc degeneration	117 (97.5)
L1-L2	
Mild	75 (62.5)
Moderate	16 (13.3)
L2-L3	
Mild	70 (58.3)
Moderate	29 (24.2)
L3-L4	
Mild	72 (60.0)
Moderate	26 (21.7)
L4-L5	
Mild	70 (58.3)
Moderate	35 (29.2)
L5-S1	
Mild	55 (45.8)
Moderate	44 (36.7)
Severe	9 (7.5)
*Anatomic features*	
Five lumbar vertebrae	119 (99.2)
Transitional vertebrae (any)	25 (20.8)
Bilateral	20 (16.7)
Facet tropism (L5-S1)	5 (4.2)
Prior surgery (decompression)	1 (0.8)
Blocked vertebrae	0
Hemi-vertebrae	0
Any adjudication	102 (85.0)

L = lumbar; S = sacral; sd = standard deviation; A = anterior; P = posterior

*Ferguson’s: from middle of L3 body

**maximal slip level if > 1 vertebrae

Note: there were no surgical fusion cases

Fifty-one adults (42.5%) achieved at least 30% ODI improvement after 12 weeks of treatment. No alignment, degenerative, or anatomic factors identified in lumbar radiographs were associated with this clinically meaningful improvement in disability at 12 weeks (i.e. all *p* > 0.05), regardless of severity of radiographic findings. A subset of results for the most common radiographic findings are reported in Table 3. The association between retrolisthesis and 30% improvement in ODI was borderline but did not reach statistical significance in this sample.

**Table 3:** Unadjusted association between radiographic features and improvement in disability

Radiographic feature	Overall *n* = 120 *n* (%)	*n* (%) with factor that met 30% ODI reduction *n* (%)	Odds ratio	95% CI	*p*-value*
***Coronal***					
Scoliosis					
No scoliosis	78 (65.0)	33 (42.3)	ref	ref	ref
Scoliosis (> 10 degrees)	42 (35.0)	18 (42.9)	1.02	0.48, 2.18	0.954
***Sagittal***					
Ferguson’s weight bearing line					
Normal	44 (36.7)	19 (43.2)	ref	ref	ref
Anterior	56 (46.7)	24 (42.9)	1.03	0.50, 2.12	0.941
Posterior	20 (16.7)	8 (40.0)	0.88	0.33, 2.35	0.804
Anterolisthesis					
None	56 (46.7)	24 (42.9)	ref	ref	ref
One level	53 (44.2)	20 (37.7)	0.70	0.34, 1.47	0.348
Two levels	11 (9.2)	7 (63.6)	2.59	0.71, 9.36	0.148
Retrolisthesis					
None	76 (63.3)	37 (48.7)	ref	ref	ref
Any level(s)	44 (36.6)	14 (31.8)	0.49	0.23, 1.07	0.071
Disc degeneration (most advanced)**					
Mild	49 (40.8)	21 (42.9)	ref	ref	ref
Moderate	55 (45.8)	23 (41.8)	0.95	0.46, 1.96	0.890
Severe	16 (13.3)	7 (43.8)	1.06	0.37, 3.07	0.913
***Anatomic***					
Transitional vertebra					
None	95 (79.2)	37 (39.0)	ref	ref	ref
L5 or S1	25 (20.8)	14 (56.0)	1.995	0.82, 4.86	0.129

Ref = reference value

CI = confidence interval

*Chi square p-value from unadjusted logistic regression

**Only 3 adults did not have any disc degeneration. We used mild disc degeneration as the reference group for logistic regression and added those 3 to the 46 with mild degeneration as one group for analysis

## Discussion

Baseline individual lumbar radiographic findings were not associated with recovery from back related disability in this sample of older adults receiving 12 weeks of chiropractic spinal manipulation and home exercise. While 42% of participants did achieve 30% improvement in back-related disability, neither the presence or absence of a pre-defined set of degenerative changes or anatomic features, nor their severity, appear to have influenced this clinical outcome. Even cases with advanced radiographic changes or abnormalities were no more likely to respond to the chiropractic and home exercise treatment delivered in the study. While this research is a retrospective analysis of only a relatively small sample (*n* = 120), it adds to the growing debate over the usefulness of routine lumbar imaging for older adults with nonspecific back pain [43]. 

The American College of Radiology recommends that radiography, in addition to MRI or CT without contrast, is usually appropriate for “elderly individuals” for back pain [44]. This recommendation is made for older adults with or without radiculopathy and in the absence of evidence of trauma or other variables that give rise to the suspicion of osteoporosis or vertebral fracture. In contrast, Choosing Wisely, an initiative that aims to reduce waste in healthcare and avoid unnecessary tests and procedures, does not identify age as an absolute risk factor for imaging requirements [45]. The American Academy of Family Physicians (AAFP) recommends withholding imaging for low back pain within the first six weeks of symptom onset unless red flags are present [46]. The AAFP does not identify older age as a singular risk factor, unless associated with a minor fall, lifting injury or evidence of osteoporosis [47]. Similar to AAFP, the American College of Physicians/ American Pain Society recommend against routine imaging in patients with non-specific low back pain. They further clarify that among patients older than 50 years of age without other risk factors, delaying imaging in lieu of a standard course of care is appropriate [48]. Of note, a review of red flag indicators among 16 low back pain guidelines found inconsistencies across most red flags, including age as a frank indicator of additional clinical caution [49]. Further, in the presence of red flags, MRI or CT are recommended modes of imaging over plain film due to higher sensitivity [50]. 

Other clinical research conducted on this topic has failed to demonstrate a positive relationship between imaging and improved outcomes. A study by Jarvik demonstrated that older adults who had early imaging for an episode of new low back pain did not have better outcomes after one year compared to those with no or delayed imaging [51]. Moreover, those who received early imaging had substantially greater use of interventions and total cost of care compared to the study group that did not have earlier imaging. Ash et al. found that neither the patient nor the provider having knowledge of diagnostic imaging results impacted clinical outcomes for conservative management of acute low back pain, with the exception of general health status which improved more among those who were blinded to their imaging results [52]. Jarvik and team concluded that the value of early imaging based on age alone is uncertain despite some guidelines recommending the use of early imaging on older adults [51]. 

The literature demonstrates that common degenerative changes of the spine, likened to “grey hair or wrinkles”, do not correlate with symptoms of back pain or disability [45, 53]. Brinjikji et al. recommend that imaging findings must be interpreted in the context of the patient’s clinical condition due to high proportion of asymptomatic patients with spinal degeneration on imaging [53]. One such example is the case of lumbar spinal stenosis, a finding estimated to be present in 1 out of 5 adults over 60 years old and increases with age [54]. Notably, more than 80% of these cases are asymptomatic [54]. Recognizing the dissociation between imaging findings and clinical symptoms may be of particular importance here, as stenosis is one of the more common conditions for surgical intervention in older adults [55]. 

Osteoporosis is also common in old age, and is a safety consideration when treating older adults with manual therapy [56]. A history of osteoporosis increases the likelihood of vertebral compression fracture, underscoring the importance of a thorough account of risk factors for osteoporosis [57]. It is important to note that, if osteoporosis is suspected, radiography is not sensitive for bone loss [58]. Dual-energy x-ray absorptiometry (DXA) is the preferred course of imaging to assess bone loss [59]. As a consideration for individuals with spinal pain and osteoarthritis, a recent meta-analysis found that the frequency of osteoporosis is not greater in individuals with osteoarthritis compared to matched controls [60]. In fact, in this population where osteophyte formation is commonly associated with degenerative changes, bone mineral density can be increased in that region [60]. 

Some back pain sufferers believe that imaging is a necessary component of care [61]. Worrying and health anxiety, both of which could be either alleviated or potentiated by imaging, has been shown to increase the risk developing of long term back-related disability [62–65]. Risks of unnecessary imaging include psychological distress and fear avoidance behavior resulting from an ‘abnormal’ imaging report, as well as financial, psychological, and potential medical complications associated with follow-up testing for incidental findings [51, 66]. Imaging influences expectations for prognosis and outcome of spine care [45, 52]. As per protocol in this study, all participants who did not have recent lumbar imaging underwent x-rays to help determine inclusion and exclusion criteria. Enrolled participants received assurance that there were no clinical or radiographic indications suggesting the need for referral or that would exclude them from participating in the study. It is possible that this clinical confirmation created psychological receptivity to respond to care. Participants’ previous history of imaging, and in particular how imaging was discussed or used to inform care in the past, may have created the potential for study participants to perceive their condition as either more or less problematic, and possibly perceive themselves as either more or less likely to respond to care.

## Limitations

The parent randomized controlled trial from which this sample was taken excluded participants with significant unmanaged comorbidities, multiple lumbar surgeries, or those at high risk of adverse events with spinal manipulative therapy (e.g. severe osteoporosis). It is possible that a more inclusive sample may have resulted in otherwise not detected associations between imaging and improvement. The small sample in this retrospective study allowed only for unadjusted analyses. Given the small sample size, we examined individual radiographic features and compared improvement among those with a specific finding to those without it, rather than counts of features or a composite score based only on radiographs. However, there may be constellations of features that collectively impact outcomes (better or worse). Such an investigation was beyond the scope of this study and sample. No participant in this sample had none of the radiographic findings considered in this analysis. Retrolisthesis was the only factor that independently neared statistical significance in unadjusted testing, and could possibly be found to influence recovery in a larger sample. We are unable to report the impact of two or more levels of retrolisthesis with confidence due to the limited number of adults with this condition. Radiographs were not taken on 34% of participants in the parent RCT, due to recent lumbar imaging acquired from other healthcare facilities. In these instances, imaging reports were used in the parent trial to determine eligibility to participate but not included in this analysis due to variation in format (e.g. MRI vs. radiograph) and comprehensiveness of radiology reports. Finally, while we attempted to minimize misclassification bias by utilizing two chiropractic radiologists who independently assessed the presence or absence of specified radiographic findings, it is possible that their training and standardization led to shared misclassification of individual data points.

## Conclusion

The result of this study suggests imaging may not be helpful for predicting who will be a responder to chiropractic spinal manipulation and home exercise, and that spinal manipulation may reasonably proceed without imaging on older adult patients in the absence of red flags or suspected contraindications to care. While exploratory in nature, this secondary analysis may inform future research into the association of anatomical and degenerative changes in older adults and outcomes of care. Finally, these results can inform chiropractic education and provide prevalence estimates of radiographic changes for chiropractors who treat older adults.

## Electronic supplementary material

Below is the link to the electronic supplementary material.

Supplementary Material 1

## Data Availability

The datasets used and/or analyzed during the current study are available from the corresponding author on reasonable request.
