# Supplementary material for: The association between individual radiographic findings and improvement after chiropractic spinal manipulation and home exercise among older adults with back-related disability: a secondary analysis
Source: Chiropr Man Therap. 2025 Jan 7;33:2. doi: 10.1186/s12998-024-00566-9 (PMC11708132; doi:10.1186/s12998-024-00566-9)
Supplement: Supplementary file 1 — Supplementary Material 1 [file 12998_2024_566_MOESM1_ESM.docx]

**Appendix 1: Radiographic Variables**

Anatomic Features

1. Atypical number of lumbar vertebrae
2. Transitional Vertebrae
   1. Bilateral
   2. Transverse Process Spatulation
   3. Pseudojoint
   4. Fusion
3. Hemi-vertebrae
4. Block vertebrae
5. Prior lumbar surgery
   1. Type of surgery
      1. Decompression,
      2. Fusion (single or multilevel)
      3. Other
6. L5-S1 tropism
   1. Definition: refers to a situation where there is a difference in the orientation/angle of facet joints (i.e. between the left and right sides) with respect to each other in the sagittal plane^22^
7. Vertebral Wedging
   1. Definition: >5° difference in anterior height to posterior height of vertebral body

Disc Degeneration^23^

1. Mild: Height Loss: anterior and posterior height loss with respect to the individual height before degeneration; <33%
2. Moderate: Height Loss: anterior and posterior height loss with respect to the individual height before degeneration; >33% to <66%
3. Severe: Height Loss: anterior and posterior height loss with respect to the individual height before degeneration; >66%

Alignment

1. Scoliosis of >10 degrees^24^
   1. Rule: +/- 5 degrees is considered agreement of inter-rater reliability
2. Cobb Angle (if >10 degrees)^22,25^
   1. Decide which vertebrae are the end vertebrae of the curve deformity (the terminal vertebrae) – the vertebra whose endplates are most tilted towards each other 4.
   2. Lines are then drawn along the endplates (or the pedicles if the endplates are not properly visualized 8), and the angle between the two lines, where they intersect, is measured.

Lumbar/ Lumbosacral

1. Ferguson’s weight bearing line^26^
   1. Drawn on an erect weight bearing radiograph as plumb line drawn downwards on a sagittal image from the middle of the L3 vertebral body and usually should pass through the anterior 1/3 of the sacrum.
2. Lumbar lordosis of L1-S1^27,28^
   1. Measure the angle formed by the upper endplate of L1 and the upper endplate of S1 or the lower endplate of L5
3. Sacral base angle^29^
   1. Measured by joining a line across the plane of the superior margin of the sacrum and a horizontal line (also called lumbosacral angle, Ferguson’s angle)

Coronal and Pelvic Balance

1. Definition: Middle of L1 body plumb line aligns with S1 tubercle^30^
2. Trunk shift >2 cm to either right or left is a positive finding.

Segmental

1. Anterolisthesis^28,29^
   1. Definition: Anterior displacement (forward slip) of a vertebral body relative to the one below
2. Meyerding grade^31^
   1. 0% to 25% is a Grade I slip
   2. 26% to 50% is a Grade II slip
   3. 51% to 75% is a Grade III slip
   4. 76% to 100% is a Grade IV slip
   5. spondyloptosis > 100% is a Grade V slip
3. Wiltse-Newman Type^32^
   1. Type I (dysplastic/congenital): translation is secondary to an abnormal neural arch
   2. Type II (isthmic): translation is secondary to a lesion involving the pars interarticularis
      1. Subtype a (lytic): secondary to stress fracture, in most cases attributed to repeated extension and/or twisting motions
      2. Subtype b (elongated pars): result of multiple injury/healing events leading to elongation of the pars
      3. Subtype c (acute pars fracture): secondary to a single event and is rare
   3. Type III (degenerative): result of chronic instability and intersegmental degenerative changes
   4. Type IV (post-traumatic): fracture in a region other than the pars leading to slippage
   5. Type V (pathological): diffuse or local disease compromising the usual structural integrity that prevents slippage
   6. Type VI (iatrogenic): slip caused directly from a prior spine surgery that involved decompression of the spine without stabilization
4. Retrolisthesis^33^
   1. Backwards slippage of one vertebral body on another
   2. +/- 3mm is normal variation of retrolisthesis slip
